# Differential regulation of cytochrome P450 genes associated with biosynthesis and detoxification in bifenthrin-resistant populations of navel orangewom (*Amyelois transitella*)

**DOI:** 10.1371/journal.pone.0245803

**Published:** 2021-01-22

**Authors:** Mark R. Demkovich, Bernarda Calla, Esther Ngumbi, Bradley S. Higbee, Joel P. Siegel, May R. Berenbaum

**Affiliations:** 1 Department of Entomology, University of Illinois at Urbana-Champaign, Urbana, Illinois, United States of America; 2 Trécé Incorporated, Adair, Oklahoma, United States of America; 3 USDA-ARS, San Joaquin Valley Agricultural Sciences Center, Parlier, California, United States of America; University of Crete, GREECE

## Abstract

Pyrethroid resistance was first reported in 2013 for the navel orangeworm, *Amyelois transitella*, but the genetic underpinnings of pyrethroid resistance are unknown. We investigated the role of cytochrome P450 monooxygenases (P450s) belonging to the CYP3 and CYP4 clans using colonies derived from individuals collected in 2016 from almond orchards in two counties. One colony (ALM) originated from an almond orchard in Madera County with no reported pyrethroid resistance and the second colony (R347) originated from the same Kern County orchard where pyrethroid resistance was first reported. We used high-throughput quantitative real-time PCR (qRT-PCR) analyses of 65 P450s in the CYP3 and CYP4 clans of *A*. *transitella* to identify P450s induced by bifenthrin and associated with pyrethroid resistance. Nine P450s were constitutively overexpressed in R347 compared to ALM, including *CYP6AE54* (11.7-fold), belonging to a subfamily associated with metabolic pesticide detoxification in Lepidoptera and *CYP4G89* (33-fold) belonging to a subfamily associated with cuticular hydrocarbon (CHC) synthesis and resistance via reduced pesticide penetrance. Cuticular hydrocarbons analysis revealed that R347 produced twice as many total CHCs in the egg and adult stages as ALM. Topical toxicity bioassays for R347 determined that egg mortality was reduced at low bifenthrin concentrations and larval mortality was reduced at high concentrations of bifenthrin compared to ALM. Our discovery of both changes in metabolism and production of CHCs for R347 have implications for the possible decreased efficacy of other classes of insecticide used to control this insect. The threat of widespread pyrethroid resistance combined with the potential for cross-resistance to develop through the mechanism of reduced penetrance warrants developing management strategies that facilitate insecticide passage across the cuticle.

## Introduction

The acquisition of resistance to synthetic insecticides by agricultural pests is a continuing challenge to sustainable pest management [1]. Cytochrome P450 monoxygenases (P450s) are Phase I detoxification enzymes that catalyze oxidation reactions involving both endogenous and exogenous compounds and are critical to insect development, communication, and detoxification [2]. Cytochrome P450 monoxygenases have been extensively investigated for their roles in metabolism of xenobiotics, and their genetic diversity, broad substrate recognition, and catalytic versatility enabled insect pests to acquire resistance to multiple classes of insecticides through various mechanisms, including constitutive overexpression, mutations affecting substrate specificity, and induction through food sources [2,3].

The highly polyphagous navel orangeworm *Amyelois transitella* Walker (Lepidoptera: Pyralidae) is the most economically important pest of almonds and pistachios in California orchards. The damage inflicted is direct when larvae feed on kernels and indirect loss occurs when *A*. *transitella* facilitate contamination of aflatoxin-producing *Aspergillus* spp. [4]. The high value of these tree nut crops, representing a $7 billion industry, has led to increased insecticide applications to reduce *A*. *transitella* damage to acceptable levels [5,6]. Insecticides are sprayed for *A*. *transitella* control at hull split, when nuts are vulnerable to oviposition. Pyrethroids are the pesticides of choice for management because of their activity against other orchard hemipteran pests and their significantly lower cost relative to other insecticide families (as low as $1.00–5.00 per treated acre compared to $25.00–45.00 for other insecticides) [7,8]. *A*. *transitella* resistance to the pyrethroid bifenthrin was first reported from an almond orchard in Kern County in 2013, and a strain derived from this orchard (R347) had an 8.7- fold and 7.1-fold resistance ratio in feeding assays using bifenthrin and beta-cyfluthrin, respectively [7] Further studies aimed at identifying resistance mechanisms using synergists implicated P450 enzymes [7]. However, these studies did not identify the specific P450s directly involved in insecticide metabolism.

Insect P450s are divided into 4 clans: CYP2, CYP3, CYP4, and mitochondrial [9]. The functions of P450s in the relatively small CYP2 and mitochondrial clans, generally conserved across insect species, include biosynthesis and metabolism of steroid hormones [10]. Thus, there is limited variability in gene number and amino acid sequence of CYP2 and mitochondrial P450s [11]. In contrast, P450s in the CYP3 and CYP4 clans have proliferated as a result of duplication events enabling diversification and neofunctionalization [12]. Within Clan 3 there are multiple xenobiotic-metabolizing families involved with phytochemical detoxification and insecticide resistance, including CYP6, CYP9, and CYP321 [13–15]. *CYP6AB11*, the first P450 functionally characterized in *A*. *transitella* [16], metabolizes imperatorin, a phytochemical present in some hostplants, but there was no detection of the metabolism of the pyrethroid insecticide alpha-cypermethrin.

The role of CYP4 P450s in general is less well understood, with fewer examples connecting functions to insecticide resistance [10]. However, since 2016, several studies have confirmed the participation of CYP4G P450s in cuticular hydrocarbon (CHC) biosynthesis in the epicuticular wax layer, which in turn could mediate insecticide resistance by reducing the rate of penetrance through the cuticle [17–20]. The role of CYP4Gs was first described when Qiu *et al*. (2012) [21] demonstrated that CYP4G1 and CYP4G2 catalyze the final step of CHC synthesis in *Drosophila melanogaster* and *Musca domestica*. The functional association of CYP4Gs with insecticide resistance was demonstrated in *Anopheles gambiae*, because overexpression of *CYP4G16* and *CYP4G17* was present in a highly insecticide resistant strain possessing a thickened epicuticle that reduced the rate of deltamethrin penetration [17]. The suppression of CYP4Gs using RNA interference increased penetration and the susceptibility to insecticides in *Nilaparvata lugens* and pyrethroid-resistant *Blattella germanica* [18,19]. A mechanism that reduces penetration into the cuticle may also increase the time available for enzymes to metabolize insecticides before they reach their target sites [17–20], conferring tolerance to insecticides and contributing to the development of resistance. To date, for lepidopterans, resistance resulting from enhanced CYP4G synthesis of CHCs has been documented in only a single species, *Helicoverpa armigera* [22].

In this paper we present the results of an integrated approach to identifying mechanisms of resistance combining a gene expression screening of the complete inventory of the *A*. *transitella* P450s, a quantitative and qualitative analysis of CHC, and insecticide bioassays targeting eggs and first instar larvae We used two strains collected from almond orchards in 2016, one strain (R347) originating from the same Kern County orchard where resistance was first identified and the other strain (ALM) collected in Madera County from an almond orchard with no history of resistance. Calla *et al*. (2017) [11] established a complete *A*. *transitella* P450 inventory comprising 89 total P450s, with 65 in the CYP3 and CYP4 Clans. We conducted high-throughput quantitative real-time PCR (qRT-PCR) analysis of all P450s within the CYP3 and CYP4 clans for both strains, in order to identify transcripts inducible by bifenthrin and therefore potentially involved in resistance. We then quantified the CHC differences between these strains via gas chromatography-mass spectrophotometry (GC/MS) and conducted both feeding assays with first instar larvae to quantify oral toxicity and spray application assays targeting eggs and larvae to quantify contact toxicity.

## Materials and methods

### Insects

In 2016, the strain designated as ALM was established from larvae collected from unharvested almonds (mummies) in Madera County (by Joel P. Siegel) and a strain (R347) was established from eggs originating from larvae collected from almond mummies in the same Kern County orchards (by Brad S. Higbee) where the original 2013-R347 [7] were present and where insecticide failures had occurred. The *A*. *transitella* larvae received at University of Illinois at Urbana-Champaign were reared to adulthood on a wheat-bran based diet [23] in 500-ml Mason jars and maintained in an incubator set at 27 ± 4°C with a photoperiod of 16:8 (L:D). Adults were collected and placed in Mason jars with dry paper towels to serve as a substrate for oviposition. Eggs were collected every 48 h to provide neonate larvae for use in qRT-PCR experiments and for use in bioassays.

### Insect sample preparation for qRT-PCR experiments

All insects were reared on semi-synthetic artificial diet [24] in 28-ml (1-oz) plastic cups (Solo Cup Company, Lake Forest, IL) without insecticides until fifth instar. Within 24 h of molting into fifth instar, larvae were removed from the rearing diet and placed on the same type of artificial diet into separate cups (1 larva per cup) containing either 0.5 ppm bifenthrin dissolved in methanol or with methanol as the control solvent. After testing a range of concentrations, we determined that 0.5 ppm bifenthrin was the maximum level that maintained consistent feeding by fifth instars after transfer. Six larvae from each strain fed on control diets containing 200 μl of methanol in 5 g of artificial diet and six from each strain fed on 200 μl of 0.5 ppm bifenthrin in 5 g of diet (2.5 micrograms of bifenthrin per cup), for a total of 24 larvae. After 48 h, the midguts were dissected from all larvae and flash-frozen in liquid nitrogen.

### Sample processing

The RNA from each of the 24 samples was extracted using a Nucleospin^®^ RNA kit (Macherey-Nagel, Düren, Germany) according to the manufacturer’s protocol. The RNA was quantitated with a Nanodrop spectrophotometer (ThermoFisher Scientific,Waltham, MA), and one μg was used to synthesize cDNA with Protoscript II kit (New England Biolab, Ipswich, MA). The cDNA for each of the 24 samples was tested by end-point PCR with primers specific for the *A*. *transitella* Actin-5 (NCBI Gene ID: LOC106142213) and evaluated in a 2% agarose gel. Primers were designed to target intron-spanning transcript regions when possible, and to amplify regions between 70–120 bp. Primers for each of the target genes were designed using Primer3 software [25] as implemented in the Geneious software version 11.0.2 (Aukland, New Zealand) [26]. All primers used in a single BioMark^®^ (Fluidigm, San Francisco, CA) chip were evaluated for cross-amplification of targets using BLAST against the sequences of the full set of P450s. Primers were then evaluated with PCR in a 2% agarose gel and re-designed if necessary. Primer sequences are in S1 and S2 Tables.

### High-throughput qPCR

Two 96-well plates were prepared per run, the first containing 29 cDNA samples consisting of the cDNA synthesized from the RNA samples, a no-template control, and pooled cDNA at serially diluted concentrations of 1, 1:10, 1:100, 1:1000, and 1:10000 made up from a mixture of all the samples, as well as the 200 μM forward and reverse mixtures of primers for each of the CYP3 clan P450s and housekeeping controls (actin, EF-alpha, GADPH, Rpl32, and tubulin). The second 96-well plate contained the same 29 cDNA samples, the 200 μM forward and reverse mixtures of primers for each of the CYP4 clan P450s, and housekeeping controls (actin, EF-alpha, GADPH, Rpl32, and tubulin). These plates were submitted to the Functional Genomics Unit of the William Keck Center for Comparative and Functional Genomics at the University of Illinois at Urbana-Champaign, where a microfluidics-based qPCR was run on a Biomark^®^ 48x48 Fluidigm-Chip (San Francisco, CA) after a pre-amplification step of 15 cycles.

### Quantification of cuticular hydrocarbons in eggs and adults

Cuticular hydrocarbons were extracted from 10 clusters of 30 three-day-old eggs and from 10 individual adults, selected at random, three to five days post-eclosion (based on preliminary experiments, showing that many of the identifiable cuticular hydrocarbons were present at this age) from each strain, following the methods of Nelson and Buckner (1995) [27] with modifications [28]. Cuticular hydrocarbons were extracted by submerging individual adults or eggs for 10 minutes in 200 μl hexane (Sigma-Aldrich, St. Louis, MO) containing 25 ng per μl 1-bromooctadecane (Sigma-Aldrich, St. Louis, MO) as the internal standard; extracts were then transferred to clean glass vials. The adults or eggs were rinsed with an additional 200 μl of hexane containing the internal standard. Washed adults were visually inspected to ensure that no physical damage to the cuticle had occurred. Extracts from the rinses were added to the initial extracts and stored at -4°C until use.

### Gas Chromatography-Mass Spectrophotometry (GC-MS) analysis of cuticular hydrocarbons

GC-MS analysis of extracted CHCs was carried out on a Hewlett-Packard (HP) 6890 GC (Hewlett-Packard, Sunnyvale, CA) in splitless mode, interfaced to an HP 5973 mass selective detector (MSD), with helium carrier gas. The column was programmed from 100°C/2 min, 50°C/min to 250°C, then 250 to 320°C at 4°C/min. Injector and transfer line temperatures were 320°C. Prior to GC-MS analysis, samples were removed from the refrigerator, concentrated to dryness under a steady stream of nitrogen, and resuspended in 30 μl of hexane with internal standard from which one μl was injected into GC-MS for analysis. A control sample of hexane was run through the GC-MS every day before samples were analyzed to confirm that the GC column was clean. The adults and egg clusters were analyzed as individual replicates from each strain. Hydrocarbon peaks were identified based on their relative retention time. The abundance of each identified hydrocarbon peak was calculated relative to the internal standard.

### Egg contact toxicity assays

Eggs were selected within 24 h after turning pink–an indication that fertilization had occurred [29]. Eggs deposited on paper towels were counted under a microscope, after which the oviposition substrate paper was cut into strips containing 25 fertilized eggs per strip. Strips were cut so that eggs were evenly dispersed within strips, and only clusters of four eggs or less were used to mitigate the potential effects of larval cannibalism at high density [30]. The eggs strips were then pinned (Bioquip, black enameled—size 0) to the center of 90 mm diameter filter papers (Whatman 1004–090 Grade 4 Qualitative Filter Paper, Maidstone, United Kingdom). Each strip of eggs pinned to a single filter paper was sprayed with a 1.5-mL solution containing 1 mL water and 0.5 mL bifenthrin (in methanol) using a spray gun kit (Badger, Franklin Park, IL) at four concentrations: 5 ppm (5.0 g/ha, 2.2% field rate), 10 ppm (9.9 g/ha, 4.4% field rate), 20 ppm (19.9 g/ha, 8.9% field rate), and 40 ppm (39.8 g/ha, 17.8% field rate). The field rates were calculated based on the amount of active ingredient bifenthrin in Bifenture EC^®^ (25.1% A.I., United Phosphorous, Inc., Mumbai, India) at the maximum label rate for *A*. *transitella* control, and represent plausible field exposure rates based on our current knowledge of insecticide coverage for *A*. *transitella* [31,32]. All bifenthrin solutions were diluted from a 100 ppm stock that was stored at -20°C. Sprayed eggs pinned to filter papers were then placed inside Petri dishes (100 x 15 mm, Corning Incorporated, NY) on top of the same wheat bran diet used for rearing larvae. Prior to bifenthrin sprays, filter papers were trimmed to an 80 mm diameter in order to fit inside the 88 mm inner diameter of the Petri dishes. All sprays were repeated to include a total of ten egg strips and filter papers per concentration. Egg mortality and larval mortality were scored together for all unhatched eggs and larvae dead on the surface of filter paper at four d. Larvae that survived bifenthrin exposure in the contact toxicity assay were provided with unamended semi-defined diet for three weeks to assess delayed effects from encountering the insecticide and were counted after three weeks (25 d after exposure). A larva was recorded as “normal” if it reached the fourth or fifth instar in a time period typical of development on artificial diet in the absence of insecticide. Individuals were recorded as stunted [33] if they were only the size of a first through third instar larva after the three-week observation period. Any replicates with egg mortality greater than two standard deviations from the mean at their respective concentrations were considered outliers and removed from further analysis. Two statistical analyses were conducted; the first used the number of surviving larvae considered as normal and the second used the total number (normal and stunted) for each strain.

### Data analysis

Data collected from the Biomark^®^ platform were assessed for quality with the Real-Time PCR Analysis software (Fluidigm) utilizing a quality threshold of 0.8 (quality scores ranging from 0 to 1, with 1 being an ideal exponential amplification curve). Melting curves were also evaluated for secondary peaks for each of the 2,304 assays. To correct for fluorescence drift and other background noise, a baseline correction for the amplification curve was set utilizing the linear-derivative method (Fluidigm Real-Time PCR Analysis, Fluidigm). Ct-values were obtained by setting a qPCR cycle threshold in a by-gene basis (i.e., setting the “by-detector” option in the Fluidigm software) to account for variability between primer pairs and to allow for assays of each gene to be treated as a separate experiment. We then used SAS “Data Step” processing (SAS University Edition v. 9.4, SAS Institute Cory, NC) to process the data and SAS Proc Mixed Procedure to analyze the data starting from the obtained Ct values. The delta-Ct value (ΔCt) for each of the assays was calculated by subtracting the Ct-value for each reaction from that of the chosen reference housekeeping gene GADPH in the same sample, and this value was used for statistical analyses. One-way and multi-factor analysis of variance (ANOVA) were conducted after checking the assumption of independence by analyzing the distribution of residuals for normality, examining scatterplots of predicted vs. residual values for independence of variances, and conducting a Bartlett’s test for equal variances in the case of the multifactor ANOVA. All pairwise tests were corrected for multi-testing with the false discovery rate (FDR) method. The negative of the estimate from the t-test is equivalent to the log_2_ scaled ΔΔCt, and this measure was used to report differential expression between pairs of treatments, strains, and the combination of both factors.

A two-way ANOVA (SPSS version 24, SPSS Inc., Chicago, IL) identified differences in egg mortality, larval mortality, normal size larvae, stunted larvae, and total mortality in spray assays with bifenthrin. Total CHC counts across 10 egg clusters and 10 adults from each population were analyzed as pooled samples and tested for significance using the Student’s t-test. Two Principal Components Analyses of the egg and adult data for ALM and R347 were performed using JMP version 14.0.0 (SAS Institute, Cary, NC). The grouping obtained was confirmed using Discriminant Analysis with hierarchical clustering. Additionally, differences between ALM and R347 for the top four CHCs by mass extracted in eggs and adults were assessed using the Student’s t-test. We applied the Bonferroni Correction using *P* ≤ 0.01 as our level of statistical significance because there were five comparisons made from the same data set.

## Results

### Quantification of expression differences in ALM and R347 *A*. *transitella* strains via qPCR

The RNA from midguts of final instar larvae fed artificial diet with or without bifenthrin was extracted and subjected to microfluidics-based qPCR analysis in order to compare constitutive and inducible expression of Clan 3 and Clan 4 P450s. We excluded results from *CYP6B44v2*, *CYP6B54*, *CYP6B55*, *CYP6B56*, and *CYP6AE55* in the CYP3 clan and *CYP341J2* and *CYP341M3* in the CYP4 clan because they did not meet the quality detection threshold score of 0.8 for the amplification curve in the Fluidigm analysis software across each sample. Additionally, amplification results were highly variable in the ALM strain controls for *CYP341K1*, *CYP341J1*, and *CYP341M-*, which affected comparisons with R347 and produced constitutive overexpression differences of 84.7, 272.2, and 281.2-fold, respectively.

The levels of P450 expression in larvae that did not consume bifenthrin (control) were considered baseline indicators of constitutive expression (ALM control vs R347 control, R347 control vs ALM control) (Table 1), and the levels of P450 expression in larvae that consumed bifenthrin as indicators of induced expression—that is, upregulation or downregulation by bifenthrin in both strains (ALM control vs ALM bifenthrin, R347 control vs R347 bifenthrin) (Table 2). We chose to report all expression differences as significant when *P* ≤ 0.1 because of the stringency of the FDR correction factor. Only P450s with ≥ 2-fold changes were reported. Six Clan 3 P450s were constitutively overexpressed–four in the ALM strain and two in the R347 strain (Table 1). Ten CYP4 clan P450s were constitutively overexpressed–seven in the R347 strain and three in the ALM strain (Table 1). Of the seven P450s in R347 constitutively overexpressed in the CYP4 clan, six of them occurred as pairs within the CYP4G, CYP341, and CYP367 subfamilies. The two P450s with the highest difference in expression between strains occurred in R347 with the CYP4 clan P450s *CYP4G89* and *CYP340AJ1*, at 33.04 and 26.77-fold difference, respectively. Only two P450s were upregulated by bifenthrin, and they were both in the ALM strain–*CYP321C1v2* in the CYP3 clan and *CYP367B8* in the CYP4 clan (Table 2). Bifenthrin treatment resulted in the downregulation of multiple CYP4 clan P450s in both strains relative to their respective controls. In the ALM strain, the P450s downregulated included *CYP4AU1*, *CYP4AU2*, and *CYP4AU8*. In R347, *CYP4G89* and *CYP340AJ1* were dramatically downregulated by bifenthrin, with 39.81 and 45.77-fold-decreases, respectively.

**Table 1:** Differences in cytochrome P450s constitutively overexpressed in the midguts of larvae that fed on control (methanol) artificial diets in strain ALM and strain R347 of *A*. *transitella*.

Strain	Clan	Gene	Fold-change	*t*-statistic	DF	*P*-value
**R347**	**CYP3**	CYP6AE54	11.72	6.03	19	<0.01
		CYP6AN17	8.83	3.90	20	0.05
	**CYP4**	CYP4G89	33.04	2.78	20	0.05
		CYP4G170	5.13	3.63	20	0.03
		CYP340AJ1	26.77	2.69	20	0.06
		CYP341S1	4.08	3.49	20	0.04
		CYP341T1	18.72	2.88	20	0.05
		CYP367B8	3.25	4.20	20	0.02
		CYP367A1	2.78	2.73	20	0.05
**ALM**	**CYP3**	CYP6AB40	20.92	4.07	20	0.04
	**CYP4**	CYP4AU1	2.47	3.02	20	0.04
		CYP4AU2	2.01	3.42	20	0.03
		CYP4AU8	2.12	3.76	20	0.03

Differences were assessed through one-way and multi-factor ANOVAs followed by a series of pairwise t-tests. Significance values from pairwise comparisons were corrected through the FDR method and are reported as significant when P ≤ 0.1.

**Table 2:** Differences in cytochrome P450 expression in midguts of larvae that fed on artificial diets containing 0.5 ppm bifenthrin in strain ALM and strain R347 of *A*. *transitella*.

Response	Strain	Clan	Gene	Fold-change	*t*-statistic	DF	*P*-value
Upregulated	ALM	CYP3	CYP321C1v2	5.12	3.14	20	0.08
		CYP4	CYP367B8	2.00	2.46	20	0.08
Downregulated	ALM	CYP4	CYP4AU1	-3.13	3.81	20	0.03
			CYP4AU2	-2.40	4.28	20	0.02
			CYP4AU8	-2.47	4.51	20	0.02
	R347	CYP4	CYP4G89	-39.81	2.93	20	0.05
			CYP340AJ1	-45.77	3.13	20	0.04

Differences were assessed through one-way and multi-factor ANOVAs followed by a series of pairwise t-tests. Significance values from pairwise comparisons were corrected through the FDR method and are reported as significant when *P* ≤ 0.1.

### Evaluation of cuticular hydrocarbon content in eggs and adults of ALM and R347 strains

**Fig 1:**
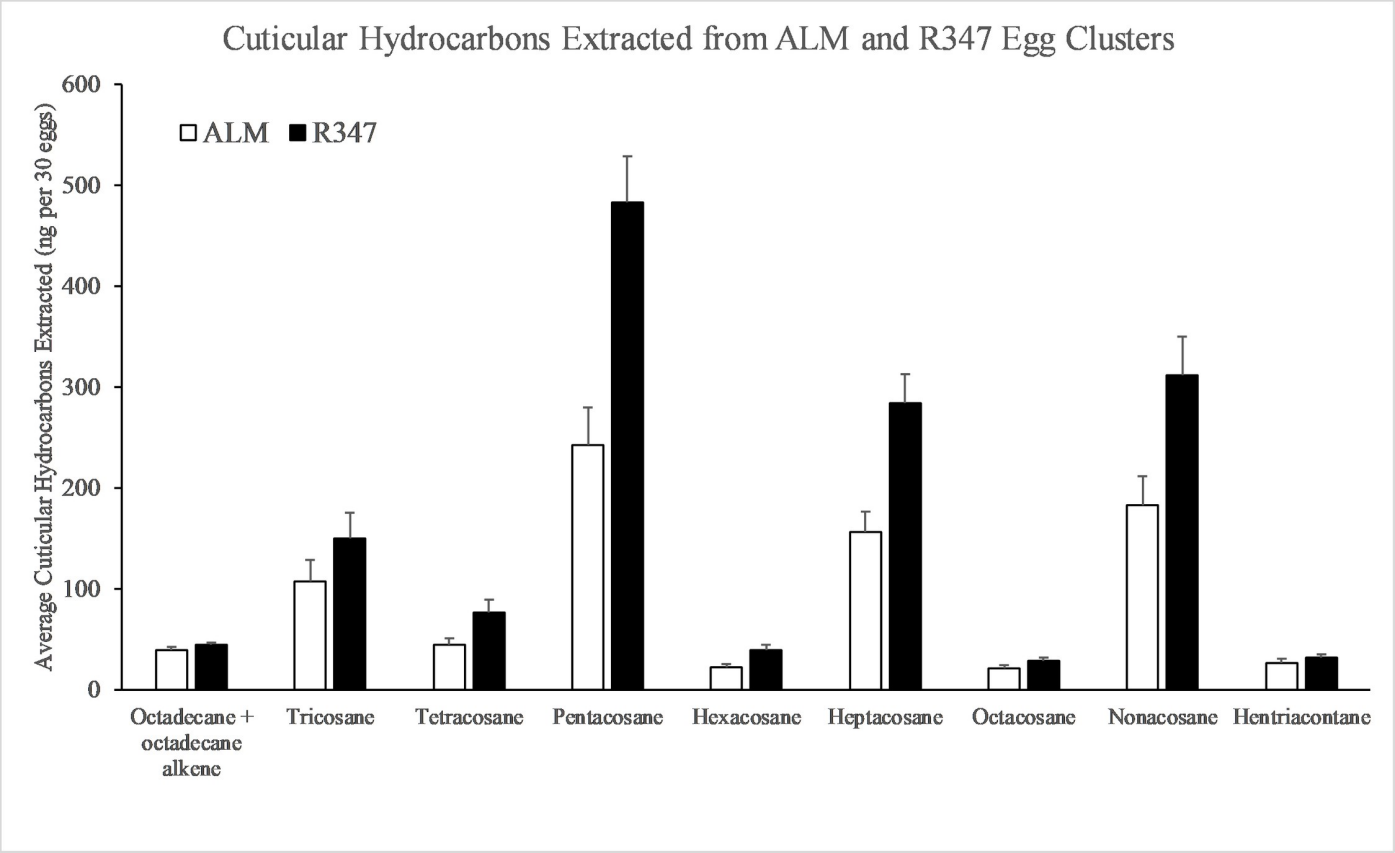
Cuticular hydrocarbons (CHCs) extracted (ng) from ten clusters of thirty eggs in ALM and R347 strains of *A*. *transitella* (n = 10 per strain) and identified through GC-MS analysis.

**Fig 2:**
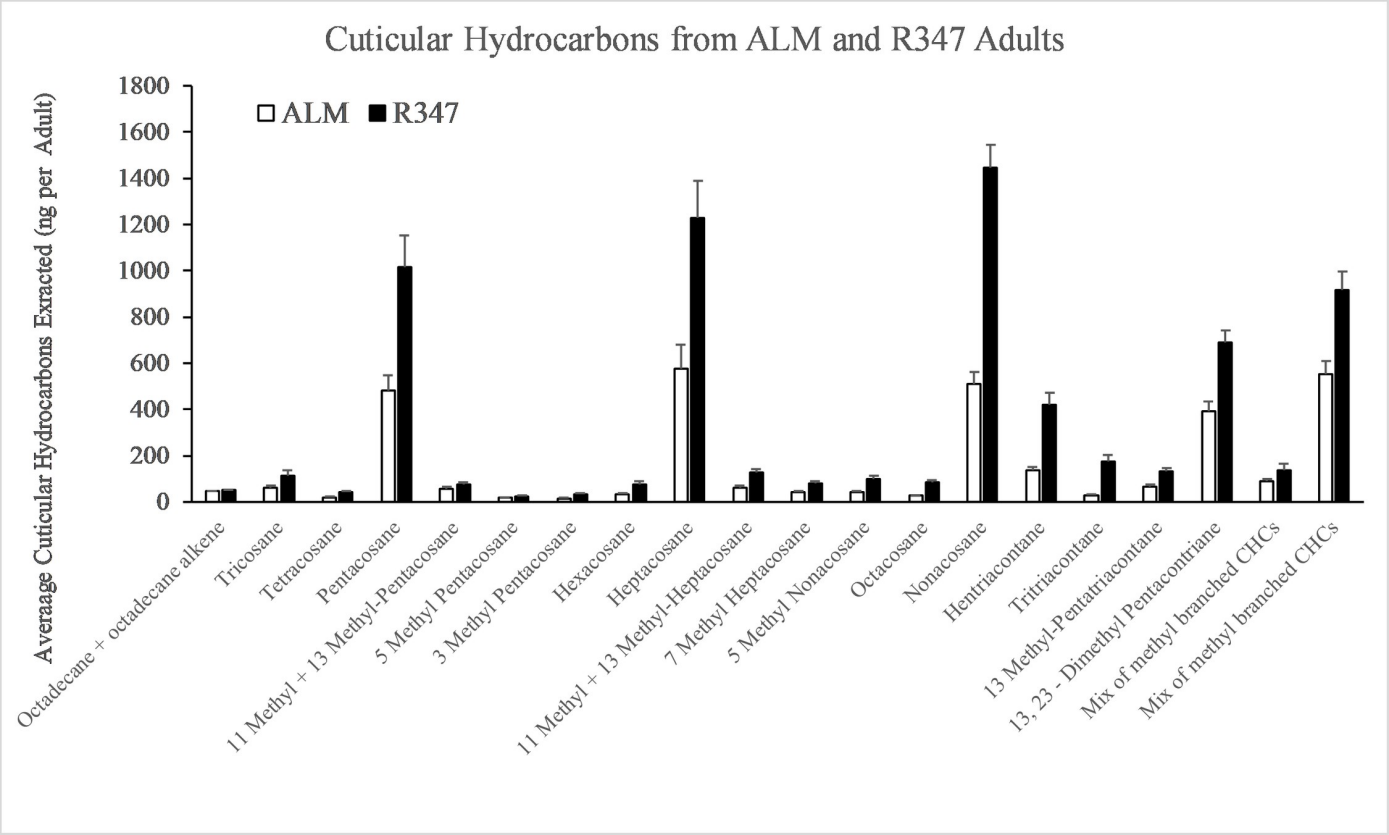
Cuticular hydrocarbons (CHCs) extracted from ten adult moths in ALM and R347 strains of *A*. *transitella* (n = 10 per strain) and identified through GC-MS analysis.

There were twice as many CHCs identified from adults as from eggs. The total CHC masses extracted were greater for both eggs and adults of R347 strain, with 1.71-fold more CHCs extracted from R347 eggs than from the ALM eggs (*t* = 3.20, df = 18, *P* = 0.005) (Fig 1), and 2.14-fold more CHCs extracted from R347 adults than from ALM adults (*t* = 5.22, df = 18, *P* < 0.001) (Fig 2). The four most prevalent CHCs from eggs were pentacosane, nonacosane, heptacosane, and tricosane, which represented 83.7% of the total CHC mass extracted (Fig 1). There was a greater mass extracted from R347 eggs than ALM eggs for pentacosane (*t* = 4.11, df = 18, *P* < 0.001) and heptacosane (*t* = 3.62, df = 18, *P* = 0.002) (Fig 1); although the mass extracted of the remaining two CHCs was numerically greater for R347 the difference was not significant at *P* ≤ 0.01. The four most prevalent CHCs from adults were pentacosane, nonacosane, heptacosane, and 13, 23 –dimethyl pentacontriane, which represented 62.2% of the total CHC mass extracted (Fig 2). There was a greater mass extracted from R347 adults than for ALM adults for all four CHCs: pentacosane (*t* = 3.63, df = 18, *P* < 0.001), heptacosane (*t* = 3.36, df = 18, *P* = 0.004), nonacosane (*t* = 8.19, df = 18, *P* < 0.001), and 13, 23 –dimethyl pentacontriane (*t* = 4.38, df = 18, *P* < 0.001).

### Principal component analyses of egg and adult samples in ALM and R347

**Fig 3:**
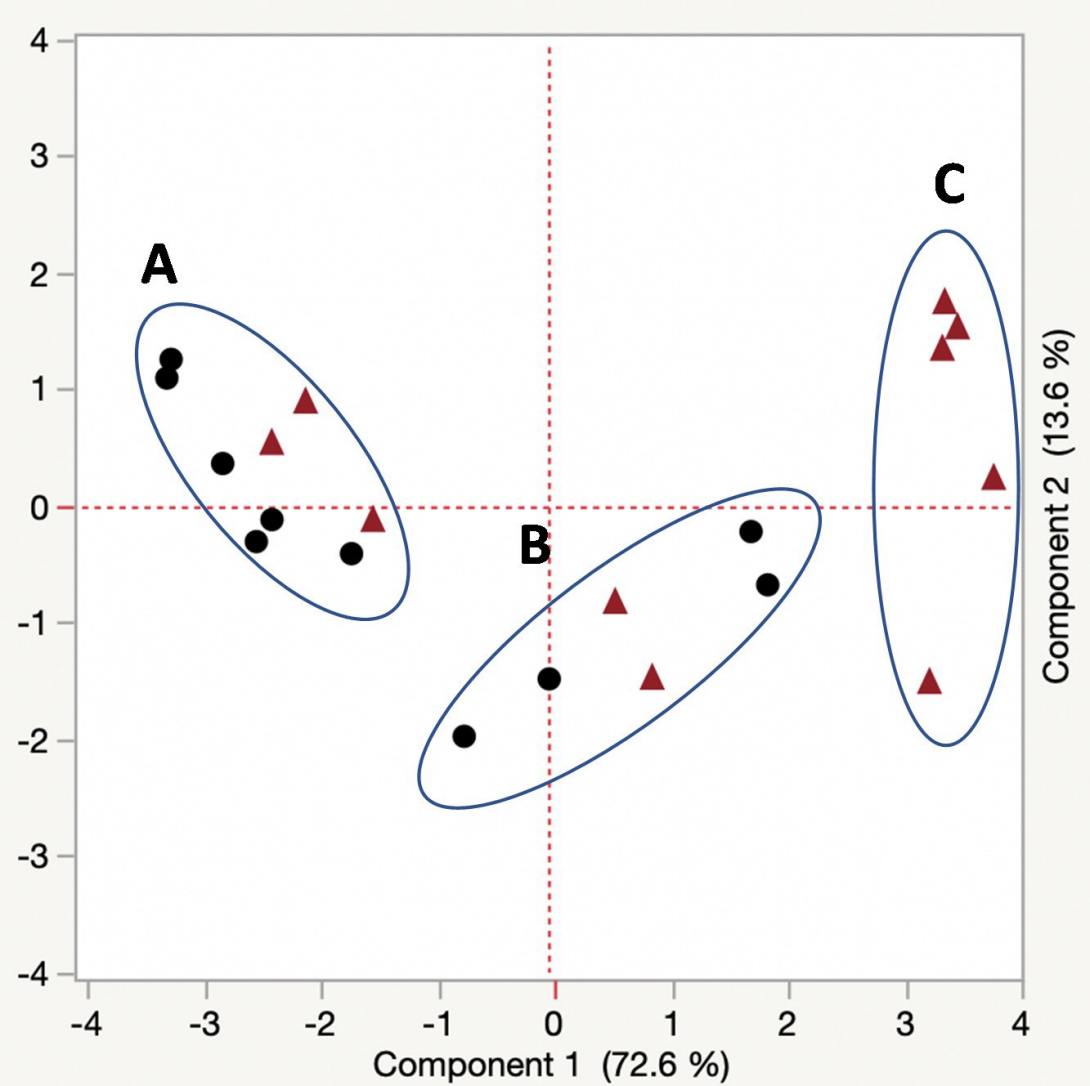
Plot of the first and second principal components of the analysis of the cuticular hydrocarbons of the egg samples (n = 10 per strain) belonging to R347 (triangle) and ALM (circle). Component 1 accounts for 72.6% of the total variation and component 2 accounts for 13.6% of the total variation. Cluster A consists of six ALM and three R347 egg samples. Cluster B consists of four ALM and two R347 egg samples, and Cluster C contains five R347 egg samples.

**Fig 4:**
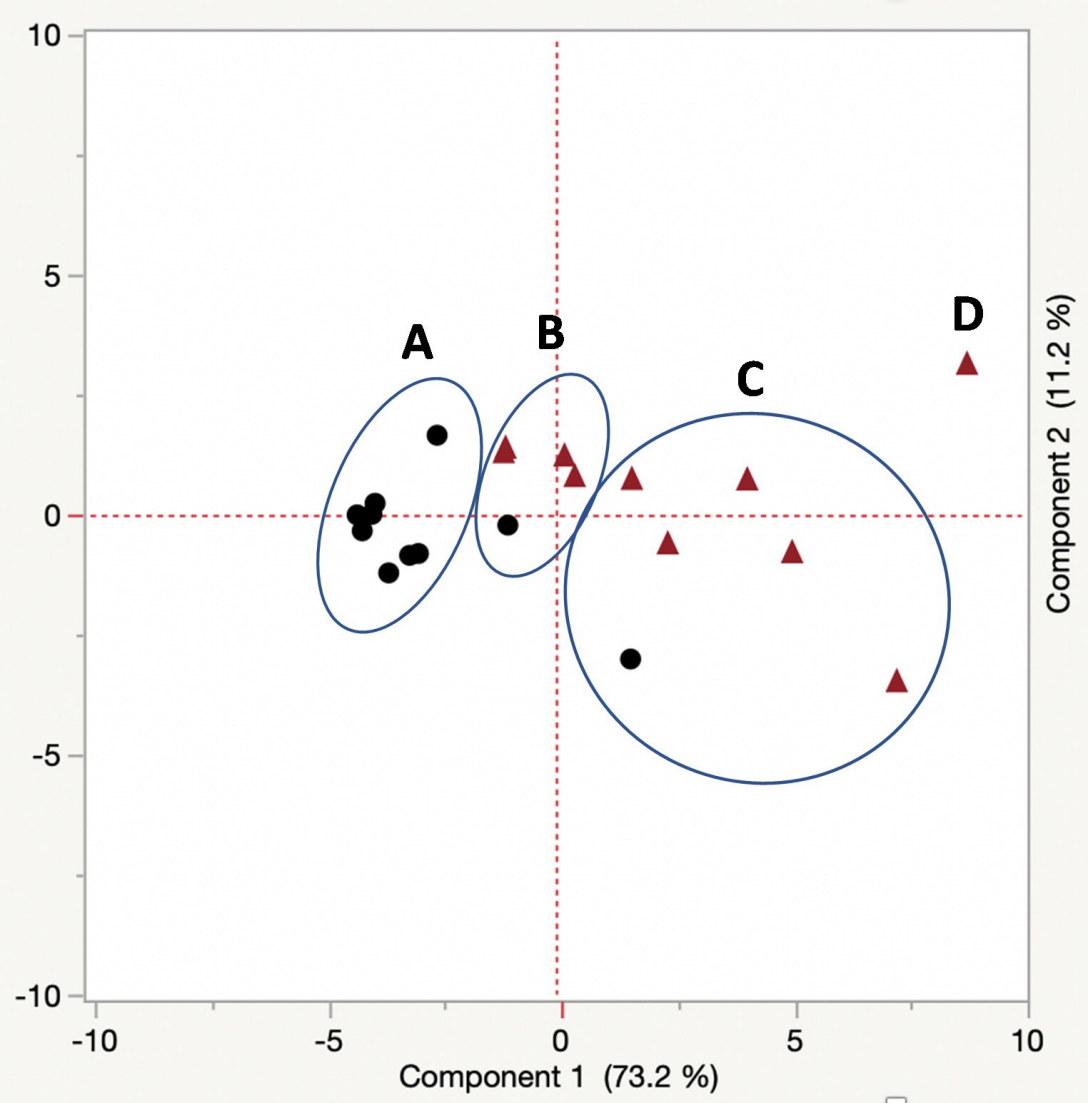
Plot of the first and second principal components of the analysis of the cuticular hydrocarbons of the adults (n = 10 per strain) belonging to strains R347 (triangle) and ALM (circle). Component 1 accounts for 73.2% of the total variation and component 2 accounts for 11.2% of the total variation. Cluster A consists of eight ALM adults, Cluster B consists of three R347 adults and one ALM adult, and Cluster C consists of five R347 and one ALM adult. There is one outlier adult marked by D; this outlier produced more cuticular hydrocarbons than any other adult from R347.

For eggs the first two principal components accounted for 72.6% and 13.6% of the total variation, respectively (Fig 3). A plot of the first two principal components revealed three distinct clusters consisting of a cluster containing three R347 and 6 ALM samples (cluster A), a cluster containing two R347 and four ALM samples (cluster B) and a homogeneous cluster containing five R347 samples (cluster C). For adults, the first three principal components accounted for 75.0% and 11.2% of the total variation, respectively (Fig 4). Strain ALM was homogeneous because eight adults clustered together (cluster A) while R347 formed two distinct clusters with each cluster containing a single ALM adult (clusters B and C). There was a single R347 adult (point D) that was separate from the two clusters of R347 but it still linked with the R347 adults.

### Egg and contact toxicity assays

We compared the contact toxicity of bifenthrin to eggs and larvae of both strains to determine whether the differential P450 gene expression and CHC production observed resulted in differences in tolerance between the two strains. The main effects of strain and bifenthrin concentration were significant for egg mortality (strain: *F* (1,99) = 6.411, *P* = 0.013; concentration: *F* (4,99) = 12.827, *P* < 0.001) (Table 3). Pairwise comparisons using Least Squares Means (LSM) revealed that R347 eggs experienced half the mortality of ALM at 5 ppm bifenthrin (*P* = 0.03) and at 10 ppm (*P* = 0.01). There were no differences between strains in contact toxicity for the larvae assessed after 4 days and there was no significant interaction.

**Table 3:** Egg and larval mortality following bifenthrin sprays on filter papers at four concentrations for strain ALM and strain R347 of *A*. *transitella*.

ALM				R347		
Concentration	*n*	% Egg Mortality	% Larval Mortality	*n*	% Egg Mortality	% Larval Mortality
Control	400	10.5 (±1.4)	0	375	10.4 (±1.6)	0
5 ppm	250	16.4 (±2.1)*	2.4 (±0.9)	250	8.8 (±1.4)*	2.0 (±0.9)
10 ppm	250	22.0 (±2.5)*	9.6 (±1.8)	225	10.7 (±2.1)*	11.6 (±2.2)
20 ppm	250	29.6 (±6.7)	21.2 (±3.4)	225	28.4 (±5.6)	16.0 (±4.8)
40 ppm	250	30.4 (±4.5)	35.2 (±3.0)	250	28.8 (±5.5)	29.2 (±5.8)

Control solution consisted of 33% methanol. One replicate was removed from R347 in the control, at 10, and at 20 ppm because the egg mortality exceeded two standard deviations from the mean. Parentheses indicate the standard error.

* Difference between ALM and R347 at respective concentrations of bifenthrin were significantly different (LSM, *P* ≤ 0.05).

### Larval survivorship three weeks after bifenthrin exposure

Survivorship did not differ between strains for the normal or stunted larvae, although the percentage of stunted larvae was approximately double for R347 at bifenthrin concentrations above 10 ppm (Table 4). Evaluation of the total number of larvae surviving after three weeks revealed significant differences between strains (*F* (1,99) = 7.722, *P* = 0.01) and across bifenthrin concentrations (*F* (4,99) = 51.525, *P* < 0.001). Pairwise comparisons using LSM showed that survivorship in R347 was almost double that of ALM at 20 ppm bifenthrin (*P* = 0.02) and 40 ppm (*P* = 0.05).

**Table 4:** Larval survivorship on bran diet following bifenthrin egg sprays on filter papers at four concentrations for strain ALM and strain R347 of *A*. *transitella*.

ALM					R347			
Concentration	*n*	% Normal	% Stunted	Total Survivorship	*n*	% Normal	% Stunted	Total Survivorship
Control	400	68.8 (±3)	4.8 (±2.1)	73.5 (±3.7)	375	65.1 (±2.1)	9.3 (±2.6)	74.4 (±2.9)
5 ppm	250	61.6 (±4.3)	4.8 (±1.8)	66.4 (±4.8)	250	63.2 (±4.8)	8.4 (±3.3)	71.6 (±4.2)
10 ppm	250	32.4 (±5.2)	12.8 (±3.8)	45.2 (±3.1)	225	35.6 (±5.2)	12.9 (±3.0)	48.4 (±5.4)
20 ppm	250	18.4 (±5.6)	8.8 (±2.8)	27.2 (±6.1)*	225	28.4 (±6.1)	16.4 (±3.4)	44.9 (±7.2)*
40 ppm	250	11.2 (±2.4)	4 (±1.7)	15.2 (±2.5)*	250	20.4 (±3.6)	7.6 (±2.5)	28.0 (±4.6)*

“Normal” larvae were fourth or fifth instars after three weeks whereas “stunted” larvae had not developed past third instar after three weeks. One replicate was removed from R347 in the control, at 10, and at 20 ppm because the egg mortality exceeded two standard deviations from the mean. Parentheses indicate the standard error.

* Difference between ALM and R347 was significant (LSM, *P* ≤ 0.05).

## Discussion

Our study is unique because we circumvented the limitation of single-gene qPCR, which becomes inefficient as the number of samples and genes increases, by screening the entire inventory of 65 CYP3 Clan and CYP4 Clan P450s in *A*. *transitella* across 24 total treatments (with a total of 2,304 assays per run). This method also proved more effective than RNA-seq, which, due to cost, limits the number of samples and treatments that can be simultaneously tested. Our results demonstrate the efficacy of applying this high-throughput method directed to a specific gene family. Although we had tested primer specificity prior to qPCR, some genes were eliminated because they failed the quality test based on the expected amplification curve; these P450s all fall into groups of very closely related sequences. We attribute this failure to either inadequate primer specificity or to unresolved/incorrect genome assembly that might have obscured gene copy number or produced other artifacts.

In our examination of the expression differences among the 65 P450s of the CYP3 and CYP4 clans, many of the P450s identified in R347 were highly expressed in larvae that fed on control diet, suggesting that constitutive overexpression is a mechanism for pyrethroid tolerance in R347. In the absence of bifenthrin, nine P450s still displayed elevated expression in R347 compared to ALM, two of which belong to the CYP3 clan and seven to the CYP4 clan. The constitutive overexpression of P450s as a mechanism for pyrethroid resistance has been observed in many insect pests across multiple orders [13,34–36]. In contrast, for the ALM strain the expression of *CYP321C1v2* was induced by bifenthrin, suggesting its involvement in bifenthrin metabolism but not resistance. Such a function is consistent with the catalytic activities of CYP321 enzymes in other polyphagous lepidopterans including corn earworm *Heliocoverpa zea* and tobacco cutworm *Spodoptera litura* [37–39]. We were surprised to detect that for both strains there were more P450s in the CYP3 and CYP4 clans downregulated by bifenthrin rather than induced by it. Although little is known about the metabolic significance of P450 downregulation, it may be a homeostatic response to toxins and divert transcriptional machinery and energy from the maintenance of normal metabolism toward the synthesis of components involved in detoxification [40].

Characterizing the function of CYP4 Clan enzymes remains elusive. In the diamondback moth *Plutella xylostella*, expression of CYP340s have been identified in larval midguts and linked to abamectin resistance, supporting the role of the family in xenobiotic metabolism [9,41]. We found that *CYP340AJ1* is constitutively overexpressed in *A*. *transitella* larvae in the absence of bifenthrin, but whether this overexpression confers tolerance to bifenthrin remains an open question. Two of the CYP4 clan P450s constitutively overexpressed in R347 belong to the CYP4G subfamily, which is linked to insecticide resistance via cuticular modification in several insect species [17–20]. The expression of multiple CYP4Gs in the midgut was unexpected, in view of the fact that CHC-synthesis has been previously associated with oenocytes in resistant insects [17,42]. In *D*. *melanogaster* and *M*. *domestica*, *CYP4G1* and *CYP4G2* encode oxidative decarboxylases as precursors to CHC synthesis, but only *CYP4G1* expression was identified in the oenocytes [21]. However, CYP4G expression may not be restricted to oenocytes because in *D*. *melanogaster CYP4G15* is expressed in the nervous system [43]. In the yellow mealworm beetle *Tenebrio molitor*, *CYP4G122* and *CYP4G123* expression was greatest in the fat body and cuticle of pupae, with the lowest expression detected in the gut [44]. Previously, RNA-seq screening in *A*. *transitella* larvae identified *CYP4G89* expression in the midgut, although at lower levels than many CYP3 Clan P450s [11,45].

Cuticular hydrocarbon production varied among strains for both life stages evaluated, and our finding is consistent with Ngumbi et al. 2020 [28], who found that for both R347 and ALM the levels of CHC varied by adult age as well as between the two strains. In our study the eggs of R347 were more homogeneous for CHC production than those of ALM because five samples clustered closely, while in contrast the adults of ALM were more homogeneous than R347 because eight clustered closely (significantly lower total CHC production). These groupings are consistent with the variable production of CHCs by both strains, but the highest production consistently occurred in the eggs and adults of R347. Despite the elevation in the levels of pentacosane, heptacosane, and nonacosane in R347, the association between these specific CHCs and insecticide resistance/tolerance is unknown. Our individual comparisons of CHCs between ALM and R347 were selected based upon total masses extracted from samples. Pentacosane, heptacosane, nonacosane, and tricosane are odd-numbered alkane CHCs, and their predominance in R347 over ALM is suggestive of a regulatory difference in biosynthetic pathways between the strains.

The detection of multiple overexpressed CYP4Gs in R347 combined with the increase in total CHCs in eggs and adults of this strain, supports the hypothesis that this overexpression acts as another resistance mechanism. More than 20 years ago elevated constitutive expression of CYP4Gs was linked to pyrethroid resistance in the lepidopteran *Helicoverpa armigera* [22] although no mechanistic explanation was provided. Balabanidou *et al*. (2016) [17] and Yahouédo *et al*. (2017) [20] measured epicuticle thickness with transmission electron microscopy to confirm differences in pyrethroid-resistant and susceptible mosquitoes, and a similar approach could help to illuminate the precise relationship between CYP4Gs, CHCs, and bifenthrin resistance in *A*. *transitella*.

In almonds, insecticides applications targeting *A*. *transitella* are primarily applied to protect the nut during hull split when the shell/kernel is exposed. Insecticide residues kill eggs directly or kill neonates when they begin feeding and as they wander across the treated hull and shell [31,32]. Our hypothesis that the cuticle of the *A*. *transitella* R347 strain has been modified, resulting in a reduction in insecticide penetration, is supported in part by our bioassays designed to simulate bifenthrin field exposure through direct exposure to spray and through contact with treated surfaces. The increased survival of R347 eggs in the bioassays suggests that they may have modifications that reduce insecticide penetration at low concentrations through the chorion. Exposure to bifenthrin spray did not differentially affect neonate mortality, but the three-week larval assessment revealed that the combination of egg and contact toxicity reduced survivorship in a concentration-dependent manner.

Insecticide resistance studies frequently omit comparisons of egg mortality relative to that of host-damaging life stages. Although insecticide resistance has been documented in the eggs of insect pests [46,47], the mechanisms contributing to enhanced survival of resistant eggs are largely unknown. Differences in egg susceptibility in this study may be due to variations in the chorion that facilitate the uptake of oxygen/or and reduce penetration of insecticides. The increased survival of R347 eggs in our bioassays at low insecticide concentration suggests that modifications to the chorion reduced insecticide penetration. However, insecticide susceptibility is also influenced by age and changes during embryonic development [48]. Because our focus was on recently fertilized *A*. *transitella* eggs, older eggs may differ in their bifenthrin tolerance.

An additional mechanism for pyrethroid tolerance/resistance is present in both strains. Calla et al. 2020 [49] identified a selective sweep in both R347 and ALM indicating that these strains contain a single point mutation in the *para* gene encoding the voltage-gated sodium channel, conferring target-site insensitivity to pyrethroids. This mutation conferred more than a 30-fold higher tolerance to bifenthrin compared to a laboratory baseline strain lacking it, and the selection pressure from heavy bifenthrin use in tree nuts from 2006 to the present may have facilitated its establishment throughout the San Joaquin Valley and perhaps the Sacramento Valley as well. The presence of this mutation in both strains may help explain their tolerance to bifenthrin when compared to the baseline strain lacking the mutation but does not explain the 3.3-fold higher tolerance of R347 relative to ALM.

A previous assessment of bifenthrin toxicity through a first instar feeding assay with these populations revealed that the median-lethal concentration (LC_50_) of bifenthrin for ALM and R347 were 7.5 ppm and 24.3 ppm, representing 3.9-fold and 12.9-fold increases, respectively, over the original LC_50_ reported for R347 in 2013 [7,49]. The ratio for R347 is now 113-fold greater than the laboratory baseline susceptible strain while for ALM, the LC_50_ of 7.5 ppm indicates that this strain is now 34.7-fold more tolerant than the laboratory baseline strain. We now consider R347 to be resistant because of the 113-fold increase in the LC_50_ compared to the original baseline but have not linked this to control failures in the field. In contrast, because the change in ALM is only 34.7-fold, we consider it to be tolerant to bifenthrin. We emphasize that bifenthrin is still effective in the field when applied properly (maximized coverage) but local *A*. *transitella* populations rebound more rapidly now after insecticide application. We purposefully chose to start colonies from populations exposed to all currently registered insecticides because it increases our credibility when suggesting changes in practice to growers, such as using the maximum label rate of insecticides and improving application efficiency through improved timing and proper sprayer speed to maintain insect control.

In conclusion, cytochrome P450s represent a single superfamily associated with xenobiotic detoxification, yet our analysis, limited to only a subset of *A*. *transitella* P450s, implicated two mechanisms that contribute to bifenthrin resistance–constitutive overexpression leading to increased detoxification combined with reduced cuticular penetrance. We acknowledge that resistance may not be limited to these routes and other detoxification mechanisms including glutathione-S-transferases (GSTs), carboxylesterases (COEs), and ABC transporters (ABCs) have been linked to pyrethroid resistance in other pest species [50–52]. The additional mechanism of changes in the target site sodium channel can also contribute to resistance. Current insecticide resistance management strategy for *A*. *transitella* depends on rotating different insecticide families, ensuring that consecutive generations are challenged by insecticides with differing modes of action. This approach, however, disregards potential overlapping mechanisms of detoxification and can be especially problematic if changes in the cuticle and/or chorion slow the penetrance of insecticides belonging to different families, despite their varying modes of action and chemical structural class [53,54]. Our finding that R347 possesses multiple mechanisms for pyrethroid resistance should be an immediate concern for growers because of the threat of cross-resistance to all registered classes of insecticides due to changes in insecticide penetrance.

## Supporting information

S1 Table Primer sequences for all CYP3 clan P450s examined in qRT-PCR experiments. (PDF)

S2 Table Primer sequences for all CYP4 clan P450s examined in qRT-PCR experiments. (PDF)

## References

[1] Brattsten LB, Holyoke CW Jr, Leeper JR, Raffa KF. Insecticide resistance: challenge to pest management and basic research. Science. 1986; 231: 1255–1260. 10.1126/science.231.4743.1255 17839561

[2] Li XC, Schuler MA, Berenbaum MR. Molecular mechanisms of metabolic resistance to synthetic and natural xenobiotics. Annu Rev Entomol. 2007; 51: 231–253. 10.1146/annurev.ento.51.110104.151104 16925478

[3] Schuler MA, Berenbaum MR. Structure and function of cytochrome P450s in insect adaptation to natural and synthetic toxins: insights gained from molecular modeling. J Chem Ecol. 2013; 39: 1232–1245. 10.1007/s10886-013-0335-7 24036972

[4] Palumbo JD, Mahoney NE, Light DM, Siegel J, Puckett RD, Michailides TJ. Spread of Aspergillus flavus by navel orangeworm (Amyelois transitella) on almond. Plant Dis. 2014; 98: 1194–1199. 10.1094/PDIS-09-13-1015-RE 30699615

[5] Administrative Committee for Pistachios in California. Pistachio Bearing Acreage, Production and Yield Per Acre [Internet]. 2018 [cited 2019 September 19]. Available from: https://acpistachios.org.

[6] United States Department of Agriculture National Agricultural Statistics Service. California Almond Objective Measurement Report [Internet]. 2019 [cited 2019 September 19]. Available from: https://www.nass.usda.gov.

[7] Demkovich M, Siegel JP, Higbee BS, Berenbaum MR. Mechanism of resistance acquisition and potential associated fitness costs in Amyelois transitella (Lepidoptera: Pyralidae) exposed to pyrethroid insecticides. Environ Entomol. 2015; 44: 855–863. 10.1093/ee/nvv047 26313992

[8] California Department of Pesticide Regulation. Pesticide Use Annual Summary Reports [Database]. 1990–2017 [cited 2019 September 19] Available from: https://www.cdpr.ca.gov/docs/pur/purmain.htm.

[9] Yu L, Tang W, He W, Ma X, Vasseur L, Baxter SW et al Characterization and expression of the cytochrome P450 gene family in diamondback moth, Plutella xylostella (L.). Sci Rep. 2015; 5: 1–9. 10.1038/srep08952 PMC5155450 25752830

[10] Feyereisen R. Insect CYP genes and P450 enzymes In: Gilbert LI, editor. Insect Molecular Biology and Biochemistry. Elsevier: Amsterdam; 2012 pp. 236–316.

[11] Calla B, Noble K, Johnson RM, Walden KKO, Schuler MA, Robertson HM, et al Cytochrome P450 diversification and hostplant utilization patterns in specialist and generalist moths: Birth, death and adaptation. Mol Ecol. 2017; 26: 6021–6035. 10.1111/mec.14348 28921805

[12] Berenbaum MR. Postgenomic chemical ecology: from genetic code to ecological interactions. J Chem Ecol. 2002; 28: 873–896. 10.1023/a:1015260931034 12049229

[13] Yang Y, Chen S, Wu S, Yue L, Wu Y. Constitutive overexpression of multiple cytochrome P450 genes associated with pyrethroid resistance in *Helicoverpa armigera*. J Econ Entomol. 2006; 99: 1784–1789. 10.1603/0022-0493-99.5.1784 17066813

[14] Hu Z, Lin Q, Chen H, Li Z, Yin F, Feng X. Identification of a novel cytochrome P450 gene, CYP321E1 from the diamondback moth, Plutella xylostella (L.) and RNA interference to evaluate its role in chlorantraniliprole resistance. Bull Entomol Res. 2014; 104: 716–723. 10.1017/S0007485314000510 25208571

[15] Ishak IH, Kamgang B, Ibrahim SS, Riveron JM, Irving H, Wondji CS. Pyrethroid resistance in Malaysian populations of dengue vector Aedes aegypti is mediated by CYP9 family of cytochrome P450 genes PLoS Negl Trop Dis. 2017; 11: e0005302 10.1371/journal.pntd.0005302 28114328 PMC5289618

[16] Niu G, Rupasinghe SG, Zangerl AR, Siegel JP, Schuler MA, Berenbaum MR. A substrate-specific cytochrome P450 monooxygenase, CYP6AB11, from the polyphagous navel orangeworm (Amyelois transitella). Insect Biochem Mol Biol. 2011; 41: 244–253. 10.1016/j.ibmb.2010.12.009 21220011

[17] Balabanidou V, Kampouraki A, MacLean M, Blomquist GJ, Tittiger C, Juárez MP, et al Cytochrome P450 associated with insecticide resistance catalyzes cuticular hydrocarbon production in Anopheles gambiae. Proc Natl Acad Sci. 2016; 113: 9268–9273. 10.1073/pnas.1608295113 27439866 PMC4995928

[18] Chen N, Pei XJ, Fan YL, Liu TX. Involvement of integument‐rich CYP4G19 in hydrocarbon biosynthesis and cuticular penetration resistance in Blattella germanica (L.). Pest Manag Sci. 2019; 10.1002/ps.5499. 31149772

[19] Wang S, Li B, Zhang D. NlCYP4G76 and NlCYP4G115 modulate susceptibility to desiccation and insecticide penetration through affecting cuticular hydrocarbon biosynthesis in Nilaparvata lugens (Hemiptera: Delphacidae). Front Physiol. 2019; 10: 913 10.3389/fphys.2019.00913 31404332 PMC6677172

[20] Yahouédo GA, Chandre F, Rossignol M, Ginibre C, Balabanidou V, Mendez NGA, et al Contributions of cuticle permeability and enzyme detoxification to pyrethroid resistance in the major malaria vector *Anopheles gambiae*. Sci Rep. 2017; 7: 1–10. 10.1038/s41598-016-0028-x 28894186 PMC5593880

[21] Qiu Y, Tittiger C, Wicker-Thomas C, Le Goff G, Young S, Wajnberg E, et al An insect specific P450 oxidative decarbonylase for cuticular hydrocarbon biosynthesis. Proc Natl Acad Sci. 2012; 109: 14858–14863. 10.1073/pnas.1208650109 22927409 PMC3443174

[22] Pittendrigh B, Aronstein K, Zinkovsky E, Andreev O, Campbell B, Daly J, et al Cytochrome P450 genes from *Heliocoverpa armigera*: expression in a pyrethroid-susceptible and -resistant strain. Insect Biochem Mol Biol. 1997; 27: 507–512. 10.1016/s0965-1748(97)00025-8 9304792

[23] Finney GL, Brinkman D. Rearing the navel orangeworm in the laboratory. J Econ Entomol. 1967; 60: 1109–1111.

[24] Waldbauer GP, Cohen RW, Friedman S. An improved procedure for laboratory rearing of the corn earworm Heliothis zea (Lepidoptera: Noctuidae). Great Lakes Entomol. 1984; 17: 113–118.

[25] Rozen S, Skaletsky H. Primer3 on the WWW for general users and for biologist programmers. Methods Mol Biol. 2000; 132: 365–386. 10.1385/1-59259-192-2:365 10547847

[26] Kearse M, Moir R, Wilson A, Stones-Havas S, Cheung M, Sturrock S. Geneious Basic: an integrated and extendable desktop software platform for the organization and analysis of sequence data. Bioinformatics. 2012; 28: 1647–1649. 10.1093/bioinformatics/bts199 22543367 PMC3371832

[27] Nelson DR, Buckner JS. The surface hydrocarbons of larval *Heliothis virescens* and *Helicoverpa zea*. Comp Biochem Physiol B. 1995; 111: 681–689.

[28] Ngumbi EN, Hanks LM, Suarez AV, Millar JG, Berenbaum MR. Factors associated with variation in cuticular hydrocarbon profiles in the navel orangeworm, Amyelois transitella (Lepidoptera: Pyralidae). J Chem Ecol. 2020; 46: 40–47. 10.1007/s10886-019-01129-6 31808076

[29] Wade WH. Biology of the navel orangeworm, Paramyelois transitella (Walker), on almonds and walnuts in northern California. Hilgardia. 1961; 31: 129–171.

[30] Bush DS, Lawrance A, Siegel JP, Berenbaum MR. Orientation of navel orangeworm (Lepidoptera: Pyralidae) larvae and adults toward volatiles associated with almond hull split and *Aspergillus flavus*. Environ Entomol. 2017; 46: 602–608. 10.1093/ee/nvx068 28379558

[31] Siegel JP, Strmiska MM, Niederholzer FJ, Giles DK, Walse SS. Evaluating insecticide coverage in almond and pistachio for control of navel orangeworm (Amyelois transitella) (Lepidoptera: Pyralidae). Pest Manag Sci. 2019; 75: 1435–1442. 10.1002/ps.5265 30430743

[32] Siegel JP, Strmiska MM, Walse SS. Evaluating insecticide coverage in almond and determining its effect on the duration of control for navel orangeworm (Amyelois transitella) (Lepidoptera: Pyralidae) in California almonds. Pest Manag Sci. 2019; 75: 2989–2995. 10.1002/ps.5413 30927307

[33] Demkovich MR, Siegel JP, Walse SS, Berenbaum MR. Impact of agricultural adjuvants on the toxicity of the diamide insecticides chlorantraniliprole and flubendiamide on different life stages of the navel orangeworm (Amyelois transitella). J Pest Sci. 2018; 91: 1127–1136.

[34] Yang T, Liu N. Genome analysis of cytochrome P450s and their expression profiles in insecticide resistant mosquitoes Culex quinquefasciatus. PLoS ONE. 2011; 6: e29418 10.1371/journal.pone.0029418 22242119 PMC3248432

[35] Liu N, Li T, Reid WR, Yang T, Zhang L. Multiple cytochrome P450 genes: their constitutive overexpression and permethrin induction in insecticide resistant mosquitoes, Culex quinquefasciatus. PLoS ONE. 2011; 6: e23403 10.1371/journal.pone.0023403 21858101 PMC3155542

[36] Zhen C, Tan Y, Miao L, Wu J, Gao X. Overexpression of cytochrome P450s in a lambda-cyhalothrin resistant population of Apolygus lucorum (Meyer-Dür). PLoS ONE. 2018; 13: e0198671 10.1371/journal.pone.0198671 29949596 PMC6021084

[37] Sasabe M, Wen Z, Berenbaum MR, Schuler MA. Molecular analysis of CYP321A1, a novel cytochrome P450 involved in metabolism of plant allelochemicals (furanocoumarins) and insecticides (cypermethrin) in Helicoverpa zea. Gene. 2004; 338: 163–175. 10.1016/j.gene.2004.04.028 15315820

[38] Rupasinghe SG, Wen Z, Chiu T, Schuler MA. *Helicoverpa zea* CYP6B8 and CYP321A1: different molecular solutions to the problem of metabolizing plant toxins and insecticides. Protein Eng Des Sel. 2007; 20: 615–624. 10.1093/protein/gzm063 18065401

[39] Wang RL, Zhu-Salzman K, Baerson SR, Xin XW, Li J, Su YJ, et al Identification of a novel cytochrome P450 CYP321B1 gene from tobacco cutworm (Spodoptera litura) and RNA interference to evaluate its role in commonly used insecticides. Insect Sci. 2017; 24: 235–247. 10.1111/1744-7917.12315 26782704

[40] Davies L, Williams DR, Aguiar-Santana IA, Pedersen J, Turner PC, Rees HH. Expression and down-regulation of cytochrome P450 genes of the CYP4 family by ecdysteroid agonists in *Spodoptera littoralis* and *Drosophila melanogaster*. Insect Biochem Mol Biol. 2006; 36: 801–807. 10.1016/j.ibmb.2006.08.001 17027846

[41] Gao X, Yang J, Xu B, Xie W, Wang S, Zhang Y, et al Identification and characterization of the gene CYP340W1 from Plutella xylostella and its possible involvement in resistance to abamectin. Int J Mol Sci. 2016; 17: 274 10.3390/ijms17030274 26999122 PMC4813138

[42] Kefi M, Balabanidou V, Douris V, Lycett G, Feyereisen R, Vontas J. Two functionally distinct CYP4G genes of *Anopheles gambiae* contribute to cuticular hydrocarbon biosynthesis. Insect Biochem Mol Biol. 2019; 110: 52–59. 10.1016/j.ibmb.2019.04.018 31051237

[43] Maїbѐche-Coisne M, Monti‐Dedieu L, Aragon S, Dauphin‐Villemant C. A new cytochrome P450 from *Drosophila melanogaster*, CYP4G15, expressed in the nervous system. Biochem Biophys Res Commun. 2000; 273: 1132–1137. 10.1006/bbrc.2000.3058 10891384

[44] Wang SY, Price J, Zhang D. Hydrocarbons catalysed by *TmCYP4G122* and *TmCYP4G123* in *Tenebrio molitor* modulate the olfactory response of the parasitoid *Scleroderma guani*. Insect Mol Biol. 2019: 10.1111/imb.12581 30843299

[45] Noble KG. Xenobiotic detoxification in the navel orangeworm (Amyelois transitella). Dissertation, University of Illinois at Urbana-Champaign. 2013. Available from: https://www.ideals.illinois.edu/handle/2142/45574.

[46] Toloza AC, Germano M, Cueto GM, Vassena C, Zerba E, Picollo MI. Differential patterns of insecticide resistance in eggs and first instars of Triatoma infestans (Hemiptera: Reduviidae) from Argentina and Bolivia. J Med Entomol. 2008; 45: 421–426. 10.1603/0022-2585(2008)45[421:dpoiri]2.0.co;2 18533435

[47] Rodríguez MA, Marques T, Bosch D, Avilla J. Assessment of insecticide resistance in eggs and neonate larvae of Cydia pomonella (Lepidoptera: Tortricidae). Pestic Biochem Physiol. 2011; 100: 151–159.

[48] Campbell BE, Pereira RM, Koehler PG. Complications with controlling insect eggs In: Trdan S, editor. Insecticide Resistance. Intech: Rijeka, Croatia; 2016 pp. 83–96.

[49] Calla B, Demkovich MR, Siegel JP, Gomes Viana JP, Walden K, Robertson HM, et al Selective sweeps in a nutshell: the genomic footprint of rapid insecticide resistance evolution in the almond agroecosystem. Gen Biol Evol. 2020; Forthcoming. 10.1093/gbe/evaa234 PMC7850051 33146372

[50] Achaleke J, Martin T, Ghogomu RT, Vaissayre M, Brévault T. Esterase-mediated resistance to pyrethroids in field populations of Helicoverpa armigera (Lepidoptera: Noctuidae) from Central Africa. Pest Manag Sci. 2009; 65: 1147–1154. 10.1002/ps.1807 19548293

[51] Labbé R, Caveney S, Donly C. Genetic analysis of the xenobiotic resistance associated ABC gene subfamilies of the Lepidoptera. Insect Mol Biol. 2011; 20: 243–256. 10.1111/j.1365-2583.2010.01064.x 21199020

[52] Carvalho RA, Omoto C, Field LM, Williamson MS, Bass C. Investigating the molecular mechanisms of organophosphate and pyrethroid resistance in the fall armyworm Spodoptera frugiperda. PLoS ONE. 2013; 8: e62268 10.1371/journal.pone.0062268 23614047 PMC3629120

[53] Niu G, Pollock HS, Lawrance A, Siegel JP, Berenbaum MR. Effects of a naturally occurring and a synthetic synergist on toxicity of three insecticides and a phytochemical to navel orangeworm (Lepidoptera: Pyralidae). J Econ Entomol. 2012; 105: 410–417. 10.1603/ec10194 22606811

[54] Demkovich M, Dana CE, Siegel JP, Berenbaum MR. Effect of piperonyl butoxide on the toxicity of four classes of insecticides to navel orangeworm (Amyelois transitella) (Lepidoptera: Pyralidae). J Econ Entomol. 2015; 108: 2753–2760. 10.1093/jee/tov237 26470383

