# Supplementary material for: Differential regulation of cytochrome P450 genes associated with biosynthesis and detoxification in bifenthrin-resistant populations of navel orangewom (Amyelois transitella)
Source: PLoS One. 2021 Jan 22;16(1):e0245803. doi: 10.1371/journal.pone.0245803 (PMC7822513; doi:10.1371/journal.pone.0245803)
Supplement: S1 Table — (PDF) [file pone.0245803.s001.pdf]

Supplementary Table 1. Primer sequences for all CYP3 clan P450s examined in qRT-PCR experiments.

P450	Forward Primer Sequence (5'-3')	Reverse Primer Sequence (5'-3')	Amplicon size (bp)
CYP6AB11v2	GAGAGAAAGCGAAATGTGGTC	TCACTTCACACAAATTGCATTTTCT	99
CYP6AB39	AAGATGGCCTAAAATAGACCCAAA	TTCTCTCGTTAAACATCAACGGC	110
CYP6AB40	CCAAGAGGCACAAGTTCGTG	AACCTGCCACTGACTGCATT	93
CYP6AB41	GTTTGGAGAGGGACCCAGAG	TCTGTAGGACAGCTGCTAGTC	83
CYP6AB42	CATAGGACCACGCGCTTGTA	CTGGCTTCACGGTGAACTTG	95
CYP6AB43	GGTGAAGGTCCTCGTTCATGT	GCGAGATAGTACTGCAGCCA	82
CYP6AB44	ATTTGGAATCGGACCACGCA	TTGACAATATGGCCGCCAAG	83
CYP6AB110v1	AGAGATGACTTACTTGGAGTGTGT	CACACTCCCGCATCAGGTAG	80
CYP6AE53	TGGATGTCATAGGATCATGTGCA	AAACGTTTCTGAGAATATCAAACAGC	110
CYP6AE54	AGAATCAAGAGGCTCAGGCA	TCATCAACACAGGCTTCCGT	121
CYP6AE55	ATGTGCATGGAGTAAGGCAT	AGAGTAGCTCTCCGATGTTTGT	135
CYP6AE56	CGGTTTGTGTGCATTTGGC	TGTATGACTCCATAAACATTATAGCTG	101
CYP6AE57	CTCTGCCTTGTGGTGCAGTA	GGTTCGGGGAAGAAGTCTGG	91
CYP6AE58	GCAGCAACAATACGTCGGAC	TGGATGCTGTACCATCAGGG	112
CYP6ML1	ATCGCTTCAAGATCGTCATCA	AGAAGGTCCCCGGAACTGTA	80
CYP6AN17	AACCGGCAGCCTTTTACGTA	ATCCCGCCAAAGACTGCATT	91
CYP6AW1	GCCCCGTCAGTCAGAGTATC	TGTGTACTATATCATGGTGCGGT	95
CYP6B44v2	ATACCTTTTGGGGTCGGACC	GCAGCTTCATCATGCACACT	82
CYP6B54	TTGTACAACAGAGCTTTCATATGAA	CACATCTTTATTCTCGACGCTCG	109
CYP6B55	TCCCTACCGAGTCGTTACCA	ACCATACTGAATAAACCGCGGT	189
CYP6B56	TGTGCGCATATACCCTTTGGA	GCTAAACAAATGCGACTCTGC	83
CYP6CV3	TCTGCCTTTTGGAGACGGAC	GAAATGCGGCCATTCCCATC	83
CYP9A63	ACACGGAATTTCGAGAGGGTC	CCAGCTCCACGTCTCTGATC	100
CYP9A64	GCAAGCGGATCCTTTCTTCG	TGAACGCTGGACTCAAGGTC	89
CYP9A65	TGCTCCCGTTCATGACAGAG	GATGACGTCATTGGCGTAGC	131
CYP9A66	CCAGATGGTGATGACCCTCA	CGCGATGACGTCATTTGTACA	106
CYP9G13	ATTGTGACGTTCGTGGTTGC	ATAGGCACCCCGGGTACATA	98
CYP9G30	ATGTCTATGGTGCTAGCAATCCT	TCCACGACATCCTCTGGGAA	80
CYP321C1v2	ACGGACAAGCCAGTGTTTCA	GAAGTTTCCAAAGCCGACGG	81
CYP321C3	GCATAAGACTGCTGCCTCCT	TGCCACAAATACTTTAGTCCCC	110
CYP321C4	GCTGTGTTTGGAGTGGAATCAG	AACGTAGATTTGAAAGCTCGTGT	80
CYP321C12v1	ACTCTTTTTACCACATGCCCG	CCGGGTCTAAAGCCAGGAAG	94
CYP321C12v2	GGCTTCATGACTCCAACGCT	GATCTCGGTCCCTCGTTGAC	99
CYP321F6	ACGTTTTTCACACCGTCCCT	ATCTCGAACCCTCTCTCGCT	98
CYP324A11	TGGGGAAGTCTCACGTTTCTG	GCCGCCAGAACAACCAGATA	106
CYP324A27	CGAAGCGATGTTAAAGCCGG	GCTGTCTACCCCAAAAGAGG	99
CYP18A1	AGAGGAATTCACTGGACGGC	TGTAAGAAGCGTCGCTGGTT	108
CYP333A1	GAGATGCTGCTCGCTGGTAT	TCGCGAAGTTTCTCCTGCTT	98
CYP354A11	TTCAAAGGAAACCAATTCGGAGG	GCTTTTATGAGCTCTGGGTCC	80
CYP365A1	TACCGCAGACGCCATAGTTC	AGAGCCACTGCAAAGGGATC	84
