# Supplementary material for: Differential regulation of cytochrome P450 genes associated with biosynthesis and detoxification in bifenthrin-resistant populations of navel orangewom (Amyelois transitella)
Source: PLoS One. 2021 Jan 22;16(1):e0245803. doi: 10.1371/journal.pone.0245803 (PMC7822513; doi:10.1371/journal.pone.0245803)
Supplement: S2 Table — (PDF) [file pone.0245803.s002.pdf]

S2 Table. Primer sequences for all CYP4 clan P450s examined in qRT-PCR experiments.

P450	Forward Primer Sequence (5'-3')	Reverse Primer Sequence (5'-3')	Amplicon size (bp)
CYP4AU1	CCGACCGCTTTCTTCCTGAA	CAGCATGCCGAAATGACGTC	104
CYP4AU2	CACTGCGTGCACTCTGGATA	CAGGTATGGCGTATCGGGTG	85
CYP4AU8	TACCTGGGTGTGATTGGGGA	CATCCATCCGCTCACCCATT	148
CYP4G88	ACACAACTCCTTGACATCATCCA	TGGTTTCACTTAGGACGGAAGG	103
CYP4G89	TCTGGCACTAGGGAAAACGC	TTCCAAACCAAATGCGAGCG	92
CYP4G170	TTTTCAAACCTTGGCTGGGC	CGTTGGCGCTATTAATTTCCGA	83
CYP4L26	TCGAACCCCAACTTTGCAGA	AGTCCATAACCAAGCCAGGG	98
CYP340AJ1	GCCGCCCTAGAAGCTGTATT	CAACGAGCGGTCCAAACAAG	96
CYP340Q10	TACCTGATAACCCCAACGCG	TCATTGACATGTAGGCGTAGGA	84
CYP341A26	GTTTGTGGCCCTTTGTCAGC	TTGGGGTTCGATTGAGCGTT	88
CYP341J1	CCTGATCAGCGCGTGGAT	GCACACCTTCATCACCACCT	82
CYP341J2	TGGGATCGTGAGCATATGGT	ACTCCCTGATCCGACGAGAT	139
CYP341J3	AGGTCACGGGATACTCTATGGA	AGGATCGGGGCACTGGAT	96
CYP341K1	TTTTTGGCGTTCAGTTGCGG	GCAGGATATCGCAGTTTTGGT	81
CYP341L1	ACTTTATCCGCCTGTGCCAG	CGTCGGGCACAAGTTTGAAA	83
CYP341M2	TAGACTTTTCACCCGCACGA	TGAGACAGCAGAGCAGACAC	87
CYP341M3	CGGAAGGTGGGGTCTAAACA	CCGTTAGTGATAGCGTGGTCA	119
CYP341S1	CCTTTCAGTAACGGTGCAAGG	CGTCTGATGATTTGTGCCATGA	86
CYP341S2	CTGAGATAATACAATCAAAAAGACTCA	AGGTTTAGATATTTCTTTTTCACA	98
CYP341T1	CTCGAGGGCCCGTTGAAA	AGCAAACTGAGACCCAACACA	81
CYP367A1	CAGTCCAACATTTCACGGCG	CGTGACACTTGGATCTTTGCTC	83
CYP367B8	TGATCCTGACTGACCCCGAT	CCCCGAGCACCTCATACATA	99
CYP4M35	GTTTGAGGGACACGATACGACT	TGTCCTGTTTGTCCCTGTTGTT	80
CYP4M36	CGCCATCCTTATTCATACATACCG	CTGACTTCATTTCCATCATTGCG	85
CYP4M37	ATTAGACCGTGGTTGAAAGAAGG	GCTGGTGTTAAAATCTTTCTTCGC	80
CYP341M1	GGCAGTGTTCCAGAGTTATCCT	GTGGACATCATGGAGAACTGGA	86
CYP341M--	GCTTACACTGTGGACATAACTGG	TGATACCGTTGATGGCCAGT	100
